# Novel multiplex technology for diagnostic characterization of rheumatoid arthritis

**DOI:** 10.1186/ar3383

**Published:** 2011-06-24

**Authors:** Piyanka E Chandra, Jeremy Sokolove, Berthold G Hipp, Tamsin M Lindstrom, James T Elder, John D Reveille, Heike Eberl, Ursula Klause, William H Robinson

**Affiliations:** 1 Division of Immunology and Rheumatology, Department of Medicine, Stanford University School of Medicine, Stanford, CA 94305, USA; 2 Geriatric Research Education and Clinical Centers, Palo Alto VA Health Care System, 3801 Miranda Avenue, Palo Alto, CA 94304, USA; 3 Roche Diagnostics GmbH, Nonnenwald 2, 82377 Penzberg, Germany; 4 Department of Dermatology, University of Michigan, Ann Arbor, MI 48109, USA; 5 Division of Rheumatology, The University of Texas Health Science Center at Houston, Houston, TX 77030, USA

## Abstract

**Introduction:**

The aim of this study was to develop a clinical-grade, automated, multiplex system for the differential diagnosis and molecular stratification of rheumatoid arthritis (RA).

**Methods:**

We profiled autoantibodies, cytokines, and bone-turnover products in sera from 120 patients with a diagnosis of RA of < 6 months' duration, as well as in sera from 27 patients with ankylosing spondylitis, 28 patients with psoriatic arthritis, and 25 healthy individuals. We used a commercial bead assay to measure cytokine levels and developed an array assay based on novel multiplex technology (Immunological Multi-Parameter Chip Technology) to evaluate autoantibody reactivities and bone-turnover markers. Data were analyzed by Significance Analysis of Microarrays and hierarchical clustering software.

**Results:**

We developed a highly reproducible, automated, multiplex biomarker assay that can reliably distinguish between RA patients and healthy individuals or patients with other inflammatory arthritides. Identification of distinct biomarker signatures enabled molecular stratification of early-stage RA into clinically relevant subtypes. In this initial study, multiplex measurement of a subset of the differentiating biomarkers provided high sensitivity and specificity in the diagnostic discrimination of RA: Use of 3 biomarkers yielded a sensitivity of 84.2% and a specificity of 93.8%, and use of 4 biomarkers a sensitivity of 59.2% and a specificity of 96.3%.

**Conclusions:**

The multiplex biomarker assay described herein has the potential to diagnose RA with greater sensitivity and specificity than do current clinical tests. Its ability to stratify RA patients in an automated and reproducible manner paves the way for the development of assays that can guide RA therapy.

## Introduction

Rheumatoid arthritis (RA) is a systemic inflammatory condition characterized by polyarthritis of presumed autoimmune etiology. Although the production of autoantibodies against synovial antigens and an increase in cytokine levels are known to be associated with RA [1,2], the molecular basis of the disease remains unclear. Insight into the pathogenesis of RA -- and hence effective treatment of RA -- has been impeded by the heterogeneity of the disease. Not only can the disease course range from mild and self-limiting to severe and progressive, but also some patients respond well to early therapeutic intervention whereas others do not [3]. Therefore, there is a need for tests that can diagnose early-stage RA, as well as tests that can predict which RA patients will require and respond to anti-rheumatic therapies.

Diagnostic tests currently used in the management of early-stage RA are not sufficiently accurate, largely because they are based on detection of single biomarkers that are either not specific to RA, e.g. rheumatoid factor (RF) and C-reactive protein (CRP), or are present in only a subset of RA patients, e.g. autoantibodies that recognize cyclic citrullinated peptides (CCP). Even when they correctly diagnose RA, current tests cannot adequately predict the course of the disease or the response to therapy because detection of a single biomarker cannot differentiate between the multiple, distinct subtypes of RA. Simultaneous analysis of multiple biomarkers may be more informative, yielding 'biomarker signatures' of RA subtypes. Indeed, we previously demonstrated that multiplex analysis of biomarkers in early-stage RA could define molecular subtypes of RA that correlated with clinically identifiable RA subtypes [1,2]. Notably, the presence of autoantibodies targeting citrullinated proteins correlated with an increase in expression of proinflammatory cytokines [2]. In addition, we recently identified a biomarker signature of autoantibody specificities and cytokine levels that could distinguish between RA patients who will respond to anti-TNF treatment and those who will not [4].

Translation of these multiplex biomarkers onto a highly reproducible, automated platform is necessary for their use in robust validation studies and, ultimately, clinical practice. In this study, we developed such a highly reproducible, automated, multiplex biomarker assay and tested its performance in the diagnosis of RA and in the molecular stratification of RA patients into clinically relevant subtypes.

## Materials and methods

### Roche multiplex automated assay

**Figure 1:**
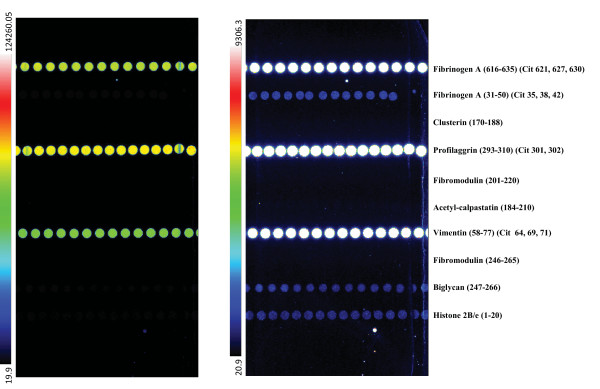
**Chips used for biomarker profiling on the IMPACT platform**. **(a) **Images of an IMPACT synovial antigen chip 1 probed with sera derived from a patient with RA. Fluoresence was captured with a charge-coupled device camera and quantified by software analysis. The images are false color representations of the fluorescence signals detected. Blue represents low, green intermediate, yellow high, and white the highest levels of fluorescence. The upper chip image is enhanced in the lower image by conversion of the lowest 5% of signals to black and the top 5% of signals to white, with the color scale adjusted accordingly. The rheumatoid arthritis sample analyzed exhibits very high levels of autoantibody reactivity to fibrinogen A (616-635) (Cit 621, 627, 630), vimentin (58-77) (Cit 64, 69, 71), and profilaggrin (293-310) (Cit 301, 302)), and low levels of antibody reactivity to fibrinogen A (31-50) (Cit 35, 38, 42), biglycan (247-266), and histone 2B/e (1-20). **(b) **List of chips and their components.

Roche Professional Diagnostics (Roche Diagnostics GmbH, Penzberg, Germany) is developing a multiplex platform called IMPACT (Immunological Multi-Parameter Chip Technology) that is based on a small polystyrene chip, as previously described [5]. During manufacturing, the chip is coated with a streptavidin layer onto which biotinylated markers -- antibodies, proteins, or peptides -- are spotted in vertical rows for the duplicate analysis of samples (Figure 1). Each chip contains up to 10 different markers, and each marker is arrayed on the chip as a vertical row of 10 to 12 spots; a minimum of five spots is required for determination of the level of a specific analyte in a sample. During the assay, the arrayed markers are probed with a small volume of sample and with a digoxigenylated secondary monoclonal antibody. The secondary antibody is then detected by the addition of an anti-digoxigenin antibody conjugated to a fluorescent latex label. This label enables sensitive detection of less than 10 individual binding events in a single spot, down to fmol/L concentrations (Roche Diagnostics, Penzberg, Germany; proprietary data on file). After this final incubation with anti-digoxigenin antibody, chips are transferred to a detection unit where a charge-coupled device camera creates an image that is converted to signal intensities, and fluorescence intensity of the array features is quantified by image analysis. The IMPACT platform currently enables multiplex analysis of up to 10 analytes in a sandwich or indirect antibody assay format, requires only microliter quantities of serum samples, and is highly sensitive. The throughput of the prototype is 40 determinations per hour. One run is intended to comprise 100 single determinations, including standards and controls.

The chips and markers used in the present study are listed in Table 1; the sequences of the peptides spotted onto the chips are listed in Table S1 in Additional File 1. Autoantibody reactivities were measured in an indirect immunoassay in which candidate RA antigens were spotted onto the chips. Levels of analytes (e.g. inflammatory and bone-turnover markers) were measured in a sandwich immunoassay in which primary, capture antibodies were spotted onto the chips. All antigens and antibody pairs on these chronic inflammatory disease (CID) chips were developed by Roche Diagnostics. For measurement of RF, human anti-IgA and anti-IgM antibodies were spotted onto the chip as capture antibodies, and the RF they bound was then detected using biotinylated polymerized human IgG. Antigens on the synovial chips [see Table S1 in Additional File 1 were selected through screens performed in the laboratory of Robinson et al. [1] or our collaborators' laboratory [6]; they were then synthesized and spotted onto IMPACT chips by Roche Diagnostics. Using the appropriate chip-specific dilution buffers, we diluted the serum samples 1:10 for use in the synovial antigen 1 and 2, CID 3, and CID 4 chips, and 1:100 for use in the CID 1 chips. In the assays using the synovial antigen 1 and 2, CID 1, CID 3, or CID 4 chips, the arrayed antigens or antibodies were probed with 40 μl of diluted serum sample, washed, and then probed with 40 μl of digoxigenylated secondary monoclonal antibody. In assays using the chips containing markers of bone turnover (bone chips), the arrayed antibodies were probed with 40 μl of serum at a 1:2 dilution and then 20 μl of digoxigenylated monoclonal antibody. Standards specific to each type of chip were included in the assays using the CID 1, CID 3, CID 4, and bone chips, and levels of each analyte were calculated on the basis of the standard curves generated. Results for the synovial antigen 1 and 2 chips (for which standards have not yet been generated) were reported and analyzed as signal intensities. We minimized non-specific binding by using fragments (Fab, Fab', or Fab'2) as capture antibodies and by using proprietary buffer reagents (in addition to the standard casein, BSA, and detergents) to minimize non-specific binding to the solid phase. For the indirect immunoassays (CCP and synovial chips), a proprietary detection antibody was used that has been optimized to ensure minimal non-specific binding. Extensive evaluation revealed that diluting the sample does not significantly influence non-specific binding (data not shown).

**Table 1:** Chips and markers used on the IMPACT platform*

Chip name	Chip components	
	Antigens	Capture antibodies
***Synovial antigen chip 1***	Histone 2B/e (1-20)	
	Biglycan (247-266)	
	Fibromodulin (246-265)	
	Vimentin (58-77) (Cit 64, 69, 71)	
	Acetyl-calpastatin (184-210)	
	Fibromodulin (201-220)	
	Profilaggrin (293-310) (Cit 301, 302)	
	Clusterin (170-188)	
	Fibrinogen A (31-50) (Cit 35, 38, 42)	
	Fibrinogen A (616-635) (Cit 621, 627, 630)	
***Synovial antigen chip 2***	Histone 2A (95-114)	
	Profilaggrin (293-310) (Cit 301, 305)	
	HSP60 (287-297)	
	Serine protease 11 (433-452)	
	Osteoglycin (177-196)	
	Apolipoprotein E (277-296) (Cit 278, 292)	
	Clusterin (334-353) (Cit 336, 339)	
	COMP (453-472)	
***CID 1***		anti-CRP
		anti-IgA (for RF measurement)
		anti-IgM (for RF measurement)
***CID 3 chip 1***	Cit peptide 1	
	Cit peptide 2	
	Cit peptide 3	
	Cit peptide 4	
***CID 3 chip 2***	Cit peptide 5	
	Cit peptide 6	
	Cit peptide 7	
	Cit peptide 8	
	Cit peptide 9	
	Cit peptide 10	
	Cit peptide 11	
***CID 4***		anti-MMP 3
		anti-IL-6
		anti-S100 protein A8/A9
		anti-E-Selectin
		anti-HABP
***Bone ***		anti-PTH
		anti-βCrosslaps
		anti-Osteocalcin
		anti-P1NP

*Candidate rheumatoid arthritis antigens were spotted on the chip for measurement of autoantibody reactivities. Primary antibodies were spotted on the chip for measurement of analyte (e.g. inflammatory mediators and products of bone turnover) levels.

Cit, citrullinated; HSP 60, heat shock protein 60; COMP, cartilage oligomeric matrix protein; CRP, C-reactive protein; MMP3, matrix metalloproteinase 3; IL-6, interleukin-6; HABP, hyaluronic acid binding protein; PTH, parathyroid hormone; P1NP, procollagen type 1 amino-terminal propeptide.

### Multiplex cytokine assay

To measure cytokine or chemokine levels in sera, we used the Milliplex Map Human cytokine/chemokine kit (Millipore, Billerica, MA, USA) run on the Luminex 200 platform coupled with BioRad Bio-Plex software (BioRad, Hercules, CA, USA), according to the manufacturers' protocols. The cytokines and chemokines measured were eotaxin, fibroblast growth factor 2, granulocyte macrophage colony-stimulating factor, IL-1α, IL-1β, IL-6, IL-12 (p40), IL-12 (p70), IL-15, IL-17, IP-10, monocyte chemoattractant protein 1 (MCP-1), and TNF. To prevent RF from bridging capture and detection antibodies in the immunoassays, we added Heteroblock (Omega Biologicals, Bozeman, MT, USA) to the sera at a final concentration of 3 μg/ml (we have shown that this concentration of Heteroblock eliminates false augmentation of the readout by heterophilic antibodies [2]). Calibration controls and recombinant standards were used as specified by the manufacturer.

### Single automated assays

Roche Tina-Quant assays run on a fully automated platform (Roche/Hitachi COBRAS C system) were used for the individual, automated measurement of CRP and RF levels in patient sera. In the CRP assay, latex particles coated with monoclonal anti-CRP antibodies agglutinate with human CRP. In the RF assay, latex-bound, heat-inactivated IgG reacts with RF to form antigen-antibody complexes. Both assays use turbidimetry to determine latex agglutination, which occurs in cases of positive test results.

### Serum samples

All patient serum samples were used after obtaining informed consent from the patients and under human subjects protocols approved by the Stanford University Institutional Review Board. Samples from RA patients were obtained from ARAMIS (Arthritis, Rheumatism and Aging Medical Information System), which includes a biobank of serum samples from 793 Caucasian RA patients who were recruited by a consortium of 161 practising rheumatologists throughout the USA [1,2,7,8]. All patients met the 1987 Arthritis College of Rheumatology criteria [9] and had RA of less than six months' duration. We used a randomisation algorithm to select serum samples from 120 patients in the ARAMIS cohort. The baseline characteristics of this subgroup of patients with early RA were assessed and found to be comparable with those of the whole cohort of patients [7]. Psoriatic arthritis (PsA) samples were provided by James T. Elder and represent a mixture of different subtypes of PsA (25% RA-like, 25% mutilans, and 50% distal interphalangeal predominant disease). Ankylosing spondylitis (AS) samples were provided by John Reveille and represent a cohort of patients with active axial and/or uveal disease. Serum samples from healthy individuals were obtained from Bioreclamation, Inc (Hicksville, NY, USA). All serum samples were shipped on dry ice, stored at -80°C, and subjected to one freeze-thaw cycle before being analyzed.

In assessing the analytical precision of the IMPACT assay, we used serum samples from the REFLEX study, a phase III trial on the efficacy of rituximab on a background of methotrexate in RA refractory to anti-TNF therapy [10]. We used only samples obtained at baseline.

### Statistical analysis

Values for each marker were divided by six times the mean value obtained for that marker in the healthy control samples and then log transformed. These normalized values were analyzed by SAM (Significance Analysis of Microarrays) [11,12]. Output was sorted based on false discovery rates (FDRs) in order to identify antigens with the greatest differences in autoantibody reactivity, or cytokines with the greatest differences in concentrations, between patients with RA, patients with other inflammatory arthritides, and healthy individuals. Most of our comparisons involved high-dimensional data, and we therefore used FDR for our exploratory analyses, an analytical method that obviates the need for multiple corrections when using high-dimensional data [11]. We then used hierarchical clustering software (Cluster^® ^3.0, developed by Michael Eisen at Stanford University, Stanford, California) to arrange the SAM results according to similarities among patient samples in autoantibody specificities or cytokine levels, and Java Treeview^® ^(Java Treeview 1.1.3, developed by Alok J. Saldanha at Stanford University, Stanford, California) to graphically display the results.

To evaluate the IMPACT assay's diagnostic sensitivity and specificity, we used a subpanel of markers from the original array results -- markers identified by univariate analysis as ones that differentiate between patients with RA and patients with other arthritides. A fluorescent value three times the mean value of that obtained in healthy control samples was defined as positive because this cutoff yielded greater specificity than a cutoff of three standard deviations above the mean. Similarly, because we had fewer healthy controls than RA cases, this method provided greater specificity than did Z-normalization. We excluded RF values from the analysis when comparing RF-positive and RF-negative subgroups, and CCP values when comparing anti-CCP-positive and anti-CCP-negative subgroups.

## Results

### Analytical precision of IMPACT assays

**Figure 2:**
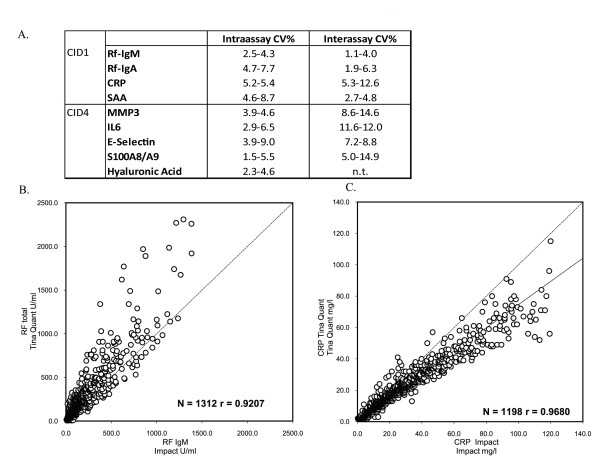
**Analytical precision of selected IMPACT assays and comparison with standard single assays**. **(a) **Analytical precision. Intra-assay coefficients of variance (CV) were generated by performing 21 replicate measurements of each of nine markers in one sample within one run on the IMPACT platform. Inter-assay CVs were calculated based on results from 5 to 15 independent runs of the same sample on the IMPACT platform. The range of the CV for each marker corresponds to that of three independent pools of sample analyzed at low, medium, and high concentrations. **(b) **Correlation of values obtained with the Roche IMPACT platform with those obtained with the standard Roche Tina Quant (latex aggregation) assay. IgM autoantibody reactivity to rheumatoid factor (IgM-RF) in 1,312 RA serum samples was measured with the IMPACT platform and with Tina Quant assay. C-reactive protein (CRP) levels in 1,198 RA serum samples were measured with the IMPACT platform and with Tina Quant assay. Linear regression was used to determine the correlation between the multiplex chip assay (IMPACT) and the standard single assay (Tina Quant). IL-6, interleukin-6; MMP3, matrix metalloproteinase 3; SAA, serum amyloid A.

To develop a system for the multiplex analysis of different types of biomarkers in the sera of RA patients, we used a bead-based commercial assay (Millipore/Luminex) to evaluate cytokine levels, and an array-based assay in development (IMPACT) to evaluate autoantibody reactivities and bone turnover. To determine the intra-assay reproducibility achieved with the IMPACT platform, we performed 21 replicate measurements of each of nine markers within one run on the IMPACT platform. The intra-assay coefficients of variance (CV) ranged from 1.5 to 9.0% (Figure 2a). To determine inter-assay reproducibility, we compared measurements obtained from 5 to 15 independent runs of the same sample at low, medium, and high dilutions; this was done for eight of the markers present on the IMPACT platform. Analysis demonstrated inter-assay CVs ranging from 1.1 to 14.9% (Figure 2a). Notably, these results compare favorably with CVs obtained with current commercial ELISA tests for RF (which yield intra-assay CVs of 6% and inter-assay CVs of 8%) [13] and CCP (which yield intra-assay CVs of 4.8 to 13% and inter-assay CVs of 9 to 17%) [14].

To assess the correlation between IMPACT multiplex assays and single automated assays, we used both the IMPACT and the Roche/Hitachi cobas c platforms to measure RF and CRP in baseline serum samples from subjects enrolled in the REFLEX study [10]. Linear regression analysis demonstrated that the correlation between the results from the multiplex assay and those from the single assay was good, with correlation coefficients of 0.92 for RF and 0.97 for CRP (Figures 2b and 2c). Analysis of the bone-turnover markers with IMPACT was previously described, the results of which correlated well with those of corresponding single automated assays [5].

### Biomarker signatures define distinct arthritides and arthritis subtypes

**Figure 3:**
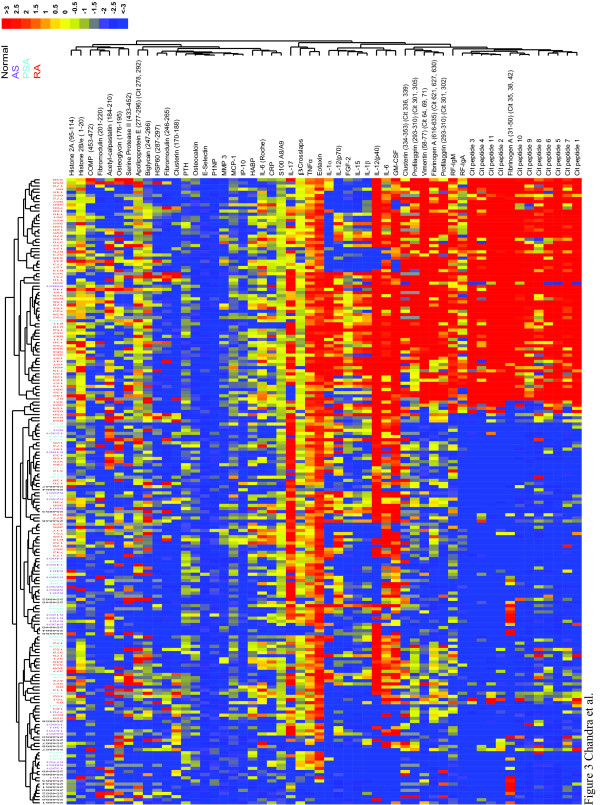
**Proteomic characterization of serum samples from patients with rheumatoid arthritis, psoriatic arthritis, or ankylosing spondylitits**. Autoantibody reactivities and levels of bone-turnover products in serum samples from 120 patients with rheumatoid arthritis (RA), 27 patients with ankylosing spondylitits (AS), 28 patients with psoriatic arthritis (PSA), and 25 healthy individuals were measured on the IMPACT platform. Cytokine levels were measured with a bead-based assay (Millipore) run on the Luminex platform. Values were normalized as described in the methods and subjected to hierarchical clustering; individual patients are listed above the heat map and the individual cytokines and antigens are listed to the right of the heat map. Cytokine levels and autoantibody reactivities are displayed, with blue representing a decrease relative to the mean value obtained in samples from healthy individuals, yellow no change, and red an increase. Cit, citrullinated; COMP, cartilage oligomeric matrix protein; CRP, C-reactive protein; FGF-2, fibroblast growth factor 2; GM-CSF, granulocyte macrophage colony-stimulating factor; HABP, hyaluronic acid binding protein; HSP 60, heat shock protein 60; IL, interleukin; MCP-1, monocyte chemoattractant protein 1; MMP3, matrix metalloproteinase 3; P1NP, procollagen type 1 amino-terminal propeptide; PTH, parathyroid hormone; RF, rheumatoid factor; TNFα, tumor necrosis factor α.

To identify molecular signatures of arthritis subtypes, we used antigen-containing chips on the IMPACT platform to measure autoantibody reactivities and bone-turnover markers [5], and bead-based assays on the Luminex platform to measure cytokines, in serum samples from 120 patients with RA, 27 patients with AS, 28 patients with PsA, and 25 healthy individuals. Values were normalized as described in the methods, subjected to hierarchical clustering, and displayed as a software-generated heat map (Figure 3). As expected, autoantibody levels were significantly higher in RA patients than in AS patients, PsA patients, or healthy controls. However, within the pool of RA patients were subgroups with distinct patterns of autoantibody specificities, including a subgroup with minimal autoantibody reactivity. Elevations in cytokine levels clearly distinguished certain subsets of patients with RA, AS, or PsA from healthy individuals. Certain subsets of arthritis patients had lower cytokine levels than did other patients with the same diagnosis. As autoantibody production is not typically a feature of PsA, the detection of autoantibodies in several patients diagnosed with PsA (Figure 3) raises the possibility that evaluation of a larger panel of autoantibodies than that measured by the commercially available assays may be able to correct misdiagnosis.

**Figure 4:**
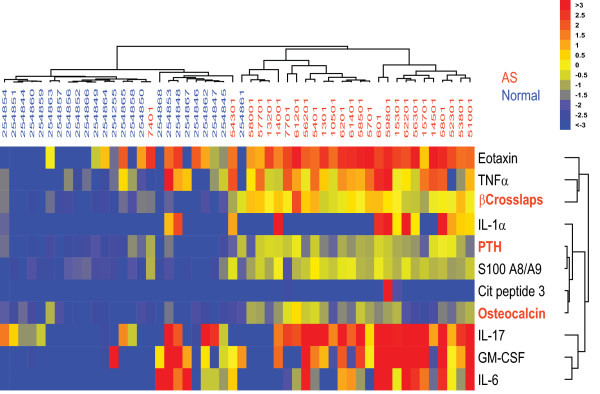
**Increased markers of bone metabolism in ankylosing spondylitis**. Autoantibody reactivity and bone-turnover products were characterized on the IMPACT platform in 27 ankylosing spondylitis (AS) patients and 25 healthy individuals. Cytokine levels in the same samples were measured using a bead-based assay run on the Luminex platform. Values were normalized as described in the methods. Significance Analysis of Microarrays (SAM) followed by a hierarchical clustering algorithm were used for determination of cluster relations that group patient samples (top dendrogram) and antigen reactivities (right dendrogram) based on similarities in patient autoantibodies and cytokines (false discovery rate < 1). Dendrogram branch lengths and distances between nodes illustrate the extent of similarities in antigen reactivity and cytokine levels, with blue representing a decrease relative to the mean value obtained in samples from healthy individuals, yellow no change, and red an increase. Bone-turnover markers are in red text. GM-CSF, granulocyte macrophage colony-stimulating factor; IL, interleukin; PTH, parathyroid hormone; TNFα, tumor necrosis factor α.

In contrast to previous findings [15,16] we did not find an association between RA and markers of bone turnover. This is perhaps not surprising given that our analysis was done using a cohort of patients with early-stage RA, and erosion of bone occurs in established and advanced RA. In contrast, an association between AS and elevated levels of markers of bone turnover -- specifically, beta crosslaps, and osteocalcin -- was revealed in the course of the biomarker analysis (Figure 4), suggesting that activation of bone-turnover pathways, exceeding that seen in RA or PsA, occurs in AS. Also intriguing was the increase in levels of the bone-marker parathyroid hormone. However, because levels of parathyroid hormone are heavily influenced by vitamin D status [17] (a variable not accounted for in our study), firm conclusions about associations between parathyroid hormone and AS cannot be drawn from our present data. Levels of proinflammatory cytokines were also significantly higher in AS patients than in healthy individuals, in line with previous findings [18,19].

### Association of biomarker signatures with parameters predictive of severe RA

Using research-grade platforms, we previously demonstrated an association between specific biomarker signatures and the presence of RF, anti-CCP antibodies, or shared-epitope (SE) alleles [1,2], each of which predicts progression to severe RA [20]. To determine whether the automated IMPACT platform could recapitulate this finding, we used the IMPACT platform in conjunction with bead-based multiplex assays to characterize serum samples from 120 RA patients, of which 73 had anti-CCP antibodies (as assessed by the IMPACT assay), 78 had RF (as assessed by the IMPACT assay), and 74 had one or two SE alleles. We performed our analysis using a subset of the antigen markers we used previously [1,2,4], as well as an additional set of analyte assays previously developed for use on the IMPACT platform (Figure 1). Data from the CCP-containing chips used to determine anti-CCP-antibody status of the patient samples (i.e., CID 3 chips 1 and 2) were excluded from analyses comparing patients on the basis of presence or absence of anti-CCP antibodies.

**Figure 5:**
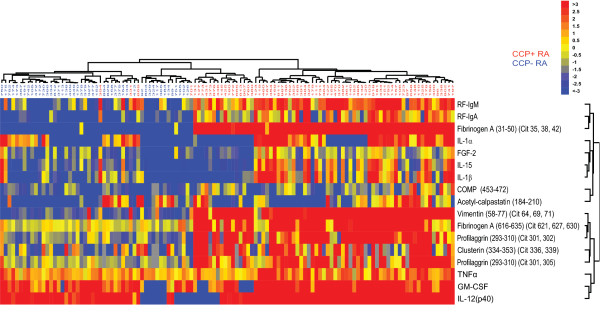
**Autoantibodies and cytokine levels stratified according to anti-CCP seropositivity**. Autoantibody and cytokine levels are higher in anti- cyclic citrullinated peptide (CCP)-antibody-positive than in anti-CCP-antibody-negative RA. Serum samples from 73 patients with anti-CCP-antibody-positive RA and from 47 patients with anti-CCP-antibody-negative RA were analyzed. Chips containing CCP were excluded from this analysis. Autoantibody reactivity was assessed on the IMPACT platform and cytokine levels were measured in a bead-based assay run on the Luminex platform. For assays run on the IMPACT platform, values were normalized as described in the methods. Significance Analysis of Microarrays (SAM) followed by a hierarchical clustering algorithm were used to determine cluster relations that group patient samples (top dendrogram) and antigen reactivities (right dendrogram) on the basis of similarities in patient autoantibody and cytokine profiles (false discovery rate < 1). Dendrogram branch lengths and distances between nodes illustrate the extent of similarities in antigen reactivity and cytokine levels, with blue representing a decrease relative to the mean value obtained in samples from healthy individuals, yellow no change, and red an increase. Cit, citrullinated; COMP, cartilage oligomeric matrix protein; FGF-2, fibroblast growth factor 2; GM-CSF, granulocyte macrophage colony-stimulating factor; IL, interleukin; RF, rheumatoid factor; TNFα, tumor necrosis factor α.

**Figure 6:**
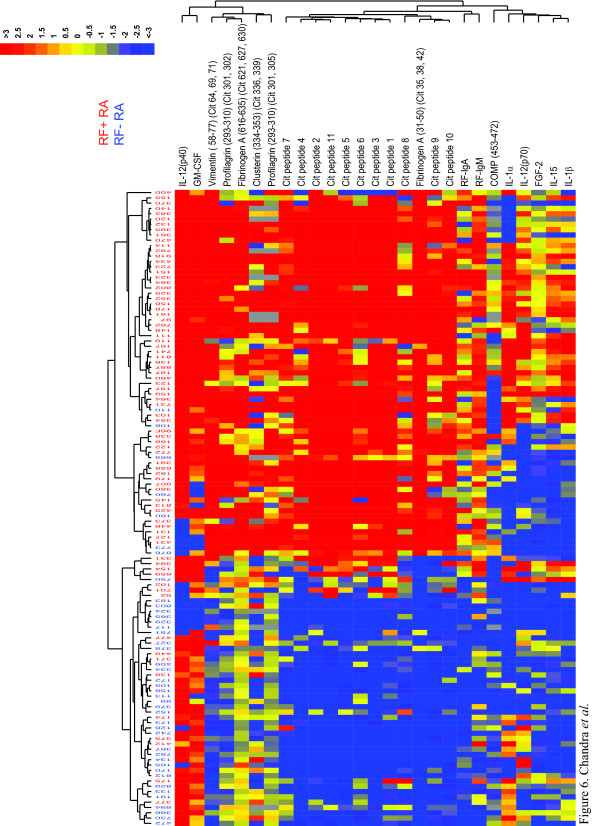
**Autoantibodies and cytokine levels stratified according to RF seropositivity**. Autoantibody and cytokine levels are higher in rheumatoid factor (RF)-positive RA than in RF-negative RA. Serum samples from 78 patients with RF-positive RA and from 42 patients with RF-negative RA were analyzed. Autoantibody reactivity was assessed on the IMPACT platform and cytokine levels were measured in a bead-based assay run on the Luminex platform. For assays run on the IMPACT platform, values were normalized as described in the methods. Significance Analysis of Microarrays (SAM) followed by a hierarchical clustering algorithm were used to determine cluster relations that group patient samples (top dendrogram) and antigen reactivities (right dendrogram) on the basis of similarities in patient autoantibody and cytokine profiles (false discovery rate < 1). Dendrogram branch lengths and distances between nodes illustrate the extent of similarities in antigen reactivity and cytokine levels, with blue representing a decrease relative to the mean value obtained in samples from healthy individuals, yellow no change, and red an increase. Cit, citrullinated; COMP, cartilage oligomeric matrix protein; FGF-2, fibroblast growth factor 2; GM-CSF, granulocyte macrophage colony-stimulating factor; IL, interleukin; RF, rheumatoid factor.

**Figure 7:**
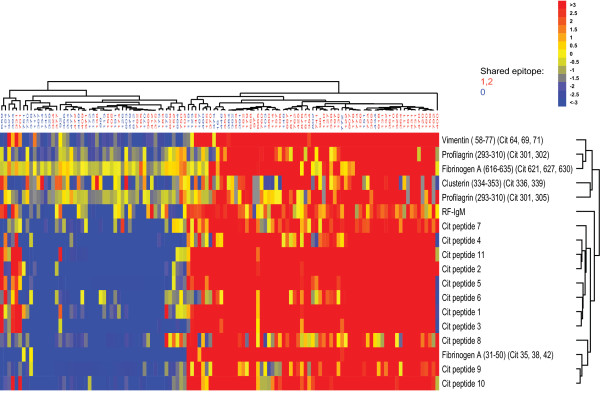
**Autoantibodies and cytokine levels stratified according to presence of a shared epitope allele**. Autoantibody levels are higher in RA patients with one or two shared-epitope alleles than in those with no shared-epitope alleles. Serum samples from 74 RA patients with either one or two copies of the shared epitope and from 46 RA patients with no shared epitope were characterized with the IMPACT platform. Autoantibody reactivity was assessed on the IMPACT platform and cytokine levels were measured in a bead-based assay run on the Luminex platform. For assays run on the IMPACT platform, values were normalized as described in the methods. Significance Analysis of Microarrays (SAM) followed by a hierarchical clustering algorithm were used to determine cluster relations that group patient samples (top dendrogram) and antigen reactivities (right dendrogram) on the basis of similarities in patient autoantibody and cytokine profiles (false discovery rate < 1). Dendrogram branch lengths and distances between nodes illustrate the extent of similarities in antigen reactivity and cytokine levels, with blue representing a decrease relative to the mean value obtained in samples from healthy individuals, yellow no change, and red an increase. Cit, citrullinated; RF, rheumatoid factor.

We again demonstrate a clear association between the presence of anti-CCP (Figure 5) or RF (Figure 6) antibodies and increased targeting of RA-associated autoantigens -- most citrullinated, but some native. Notably, distinct but overlapping sets of antigens were targeted in RF-positive patients compared with anti-CCP-antibody-positive patients. Likewise, the pattern of increases in cytokine levels showed both differences and similarities between RF-positive patients and anti-CCP-antibody-positive patients. Despite the strong association between seropositivity (the presence of RF and/or anti-CCP antibodies) and elevation of serum cytokines, a subset of seronegative patients had significantly elevated serum cytokines, possibly reflecting a subpopulation more clinically and immunologically similar to those who can be defined as seropositive. When we sought to identify differences on the basis of the presence or absence of SE alleles, we found that the presence of SE alleles was associated with increased targeting of RA-associated autoantigens; however, unlike the presence of RF or anti-CCP antibodies, the presence of SE alleles alone was not associated with elevations in serum cytokines (Figure 7). There was no significant difference between carrying one versus two copies of the SE allele (data not shown).

### Autoantibody and cytokine signatures as sensitive and specific diagnostics of RA

Using univariate analysis, we determined which of the biomarkers (out of 31 autoantigens, 4 bone markers, 5 inflammatory mediators, and 14 cytokines) distinguish RA patients from a pool of 120 patients with early-stage RA, 27 patients with AS, 28 patients with PSA, and 25 healthy individuals. We found that a panel of six autoantigens distinguished RA. We then used the same serum samples to evaluate the diagnostic sensitivity and specificity of different combinations of the individual autoantigens in this differentiating panel of six biomarkers. The sensitivity and specificity of these subpanels in the differential diagnosis of RA were similar to that of anti-CCP status [21] and better than that of RF status [22] (Table 2).

**Table 2:** Performance characteristics of multiplex-assayed autoantibody profiles in the diagnosis of rheumatoid arthritis

Number of positive biomarkers*	PPV	NPV	Sensitivity	Specificity
1+ markers	79.5%	92.6%	96.7%	62.5%
2+ markers	91.8%	89.7%	93.3%	87.5%
3+ markers	95.3%	79.8%	84.2%	93.8%
4+ markers	95.9%	61.1%	59.2%	96.3%

*Biomarker panel: histone 2B/e (1-20), vimentin (58-77) (Cit 64, 69, 71), fibrinogenA (616-635) (Cit 621, 627, 630), COMP (453-472), profilaggrin (293-310) (Cit 301, 305), RF-IgA. The cut-off value for positivity was defined as three times the mean value obtained in samples from healthy individuals. PPV, positive predictive value; NPV, negative predictive value.

## Discussion

We report the development of a highly reproducible, automated, multiplex biomarker assay that can reliably distinguish RA patients from healthy individuals or patients with other inflammatory arthritides. Multiplex measurement of a subset of the differentiating biomarkers provided high sensitivity and specificity in the diagnostic discrimination of RA. Furthermore, the biomarker profiles we identified enabled stratification of RA patients into distinct, clinically relevant subtypes.

Current clinical tests fall short of being accurate and all-encompassing diagnostics of RA because RF is not specific to RA and anti-CCP antibodies are not produced in all cases of RA. Compared with single-biomarker detection, multiplex-biomarker detection -- by casting the net wider -- provides greater sensitivity and specificity of diagnosis. Although they remain to be validated in independent cohorts of RA patients, our preliminary results suggest that our biomarker assay has the potential to provide greater diagnostic sensitivity and specificity than that provided by current clinical tests. Including in our analysis a larger number of control patients with non-RA inflammatory diseases should allow us to further increase the sensitivity and specificity of our biomarker assay. Whereas the commercial anti-CCP-antibody assay relies on the measurement of antibody reactivity against a mixture of different citrullinated peptides, our multiplex biomarker assay allows measurement of antibody reactivity against each of several different citrullinated peptides independently, thus enabling more-precise diagnostic characterization. Moreover, the integrated evaluation of multiple additional biomarkers (i.e. autoantibody specificities, cytokine levels, and bone-turnover products) enables the stratification of RA into disease subtypes and provides further insight into disease pathogenesis at the individual level. For instance, our biomarker assay identified a subset of seronegative RA patients who had elevations in serum cytokines suggestive of more aggressive disease, an association that would have gone undetected with current clinical tests.

As RA is such a heterogeneous disease, diagnosis must be accompanied by prognosis in order to identify which patients with early-stage RA are in need of aggressive therapeutic intervention. The presence of serum RF or anti-CCP antibodies is associated with progression to severe RA [23-25]. When combined, these two biomarkers offer a somewhat improved prognostic capability [26]. Although we did not observe an association of bone-turnover markers with early-stage RA in this study, elevations in markers of bone and cartilage turnover [27] also have been proposed to predict a more destructive course of RA, as have elevations in acute-phase reactants [28]. Multiplex biomarker detection should be more accurate and informative than single-biomarker detection in RA prognosis, as it is in diagnosis. We show here that multiplex biomarker detection in early-stage RA can identify biomarker signatures that are associated with immunological (presence of anti-CCP-antibodies or RF) and genetic (possession of SE alleles) parameters predictive of more severe RA. Unfortunately, information on the degree of radiographic joint damage at the time of diagnosis, a powerful predictor of disease outcome, was not available for the cohort we analyzed. In addition, the greater use of disease-modifying anti-rheumatic drugs in patients with RF-positive RA confounded attempts to correlate our biomarker signatures with disability at diagnosis (as assessed by the Stanford Health Assessment Questionaire), a good predictor of later functional impairment [29]. In addition to validating out present findings in an independent cohort of patients, we aim to evaluate the prognostic utility of our assay. Given that our biomarker panel enables disease stratification and yields detailed molecular information, we expect that it will provide more precise prognosis than that achieved in the clinic at present.

Although not an overt objective of the present study, our biomarker analysis revealed that AS is associated with elevated levels of bone-turnover markers and cytokines, in line with previous findings [18,30]. The small number of AS patients included in this study precludes any firm conclusions, but this observation suggests that our multiplex platform may be useful in developing a diagnostic or prognostic test for AS -- a major unmet clinical need.

This exploratory study has several limitations. Given that we were unable to adjust for treatment-related effects on the studied biomarkers, it is possible that use of immunosuppressant therapy could affect levels of serum cytokines and thereby confound interpretation of our data. In addition, the ARAMIS RA cohort studied represents a Caucasian, American, early-RA cohort, and therefore it is possible that our findings cannot be extrapolated to all RA patients; our findings remain to be validated in independent and more diverse cohorts. In addition, the fact that RA and control patients were not matched by demographics or by handling of their serum samples could bias our results.

## Conclusions

In the diagnosis and prognosis of RA, measurement of a single biomarker is not sufficiently sensitive or accurate, and individual measurement of multiple biomarkers is labor intensive and therefore expensive. Automated multiplex biomarker analyses can help to reduce the laboratory workload involved in the analysis of multiple biomarkers and can provide greater sensitivity and specificity. However, their use in clinical trials has been hampered by their limited reproducibility between and within multiplex platforms. The multiplex system we developed in this study is ideally suited to the simultaneous analysis of multiple biomarkers because it uses a standardized assay platform and is highly automated, allowing high-throughput reproducibility across clinical laboratories. Here we demonstrate the effectiveness of this multiplex biomarker assay in stratifying RA into clinically relevant subtypes. The ability to classify RA patients in an automated and reproducible manner paves the way for further studies aimed at attaining personalized medicine for RA.

## Abbreviations

AS: ankylosing spondylitis; CCP: cyclic citrullinated peptide; CID: chronic inflammatory disease; COMP: cartilage oligomeric matrix protein; CRP: C-reactive protein; CV: coefficient of variance; FDR: false discovery rate; IL: interleukin; IMPACT: Immunological Multi-Parameter Chip Technology; MCP: monocyte chemoattractant protein; PsA: psoriatic arthritis; RA: rheumatoid arthritis; RF: rheumatoid factor; SAM: significance analysis of microarrays; SE: shared epitope; TNF: tumor necrosis factor.

## Competing interests

BGH, HE, and UK are employees of Roche diagnostics. WHR has served on a scientific advisory board of Roche Diagnostics and has received research and reagent support from Roche Diagnostics for the study of RA biomarkers. Roche Diagnostics owns patents relating to the IMPACT platform. Stanford University has applied for patents related to RA biomarker work performed prior to this study.

## Authors' contributions

PEC, JS, BGH, HE, UK, and WHR designed the studies, performed the studies, analyzed the data, and interpreted the data. JTE and JDR provided the psoriatic arthritis and ankylosing spondylitis patient samples, respectively. TML contributed to data interpretation and provided editorial input. All authors read and approved the final manuscript.

## Supplementary Material

Additional file 1 **Sequences of peptides spotted on synovial antigen chip 1 and 2**. Supplementary table showing the amino acid sequences of the peptides spotted onto synovial antigen chips 1 and 2. Click here for file
