# Supplementary material for: Novel multiplex technology for diagnostic characterization of rheumatoid arthritis
Source: Arthritis Res Ther. 2011 Jun 24;13(3):R102. doi: 10.1186/ar3383 (PMC3218917; doi:10.1186/ar3383)
Supplement: Additional file 1 — Sequences of peptides spotted on synovial antigen chip 1 and 2. Supplementary table showing the amino acid sequences of the peptides spotted onto synovial antigen chips 1 and 2. [file ar3383-S1.PDF]

Supplementary Table 1. Sequences of peptides spotted on Synovial Antigen chip 1 and 2.

**Chip Name**	Peptide*	Peptide Sequence
Synovial Antigen chip 1	Histone 2B/e (1-20)	MPEPVKSAPVPKKGSKKAIN
	Biglycan (247-266)	EDLLRYSKLYRLGLGHNQIR
	Fibromodulin (246-265)	LEQLYMEHNNVYTVPDSYFR
	Vimentin (58-77) (Cit 64, 69, 71)	GGVYAT[CIT]SSAV[CIT]L[CIT]SSVPGV
	Acetyl-calpastatin (184-210)	Acetyl-DPMSSTYIEELGKREVTIPPKYRELLA-NH2
	Fibromodulin (201-220)	NLTALYLQHDEIQEVGSSMR
	Profilaggrin (293-310) (Cit 301, 302)	TIHAHPGS[CIT][CIT]GGRHGYHH
	Clusterin (170-188)	THMLDVMQDHFSRASSIID
	Fibrinogen A (31-50) (Cit 35, 38, 42)	GGGV[CIT]GP[CIT]VVE[CIT]HQSACKDS
	Fibrinogen A (616-635) (Cit 621, 627, 630)	THSTK[CIT]GHAKS[CIT]PV[CIT]GIHTS
Synovial Antigen chip 2	Histone 2A (95-114)	NKLLGRVTIAQGGVLPNIQA
	HSP60 (287-297)	VLNRLKVGLQV
	Serine Protease 11 (433-452)	VIISINGQSVVSANDVSDVI
	Osteoglycin (177-196)	NQLLKLPVLPPKLTLFNAKY
	Apolipoprotein E (277-296) (Cit 278, 292)	A[CIT]LKSWFEPLVEDMQ[CIT]QWAG
	Clusterin (334-353) (Cit 336, 339)	AE[Cit]LT[Cit]KYNELLKSYQWKML
	COMP (453-472)	NSAQEDSDHDGQGDACDDDD
	Profilaggrin (293-310) (Cit 301, 305)	TIHAHPGS[CIT]RGG[CIT]HGYHH

* Cit, citrullinated; HSP 60, heat shock protein 60; COMP, cartilage oligomeric matrix protein.
